# Impact of preoperative extracorporeal membrane oxygenation on vasoactive inotrope score after implantation of left ventricular assist device

**DOI:** 10.1186/s40064-015-1649-4

**Published:** 2015-12-30

**Authors:** Takuma Maeda, Koichi Toda, Masataka Kamei, Shigeki Miyata, Yoshihiko Ohnishi

**Affiliations:** Department of Anesthesiology, National Cerebral and Cardiovascular Center, 5-7-1 Fujishirodai, Suita, Osaka, 565-8565 Japan; Department of Cardiovascular Surgery, Osaka University Graduate School of Medicine, 2-2 Yamada-oka, Suita, Osaka, 565-0871 Japan; Division of Transfusion Medicine, National Cerebral and Cardiovascular Center, 5-7-1 Fujishirodai, Suita, Osaka, 565-8565 Japan

**Keywords:** Ventricular assist device, Inotrope score, Systemic inflammatory response, Vasopressor

## Abstract

The purpose of this study was to elucidate the difference in inotrope use between patients who underwent left ventricular assist device (LVAD) implantation with preoperative extracorporeal membrane oxygenation (ECMO) and those who underwent LVAD implantation without preoperative ECMO. One hundred and eight patients who underwent LVAD implantation were enrolled in this study. Prior to LVAD implantation, 27 patients received ECMO support (ECMO group) and the other 81 patients did not (non-ECMO group). Cardiac index (CI), mean arterial pressure (MAP), mixed venous oxygen saturation (SvO2), and the vasoactive inotropic score (VIS) were recorded at weaning from cardiopulmonary bypass (CPB), 30 min after weaning from CPB (min after CPB), 60 min after CPB, and at the end of surgery. MAP and VIS were also recorded before induction of anesthesia (baseline). The modified VIS was defined as: (dopamine µg/kg/min × 1 + dobutamine µg/kg/min × 1 + epinephrine µg/kg/min × 100 + noradrenaline µg/kg/min × 100 + milrinone µg/kg/min × 10 + olprinone µg/kg/min × 25). There were no significant differences between the ECMO group and the non-ECMO group in terms of hemodynamic parameters such as MAP, CI, and SvO_2_. However, the ECMO group had higher VIS and noradrenaline doses than that of non-ECMO group (*p* = 0.030 and *p* = 0.044, respectively). VIS was significantly higher in ECMO group at 30 min after CPB (*p* = 0.03), 60 min after CPB (*p* = 0.003), and at the end of the surgery (*p* < 0.001). The doses of noradrenaline were significantly higher in ECMO group at 60 min after CPB (*p* = 0.013), and at the end of surgery (*p* = 0.002). Patients who received ECMO support prior to LVAD implantation required significantly more noradrenaline to maintain normal levels of hemodynamic parameters compared with patients without ECMO.

## Background

Mechanical circulatory support with left ventricular assist devices (LVADs) is becoming increasingly important as a therapeutic intervention for patients with advanced heart failure recalcitrant to medical therapy. It is important to set up patients eligible for transplantation with appropriate hemodynamic support without delay; otherwise death or serious morbidity may occur.

Several risk factors have been identified in regard to mortality after LVAD implantation (Holman et al. 2009; Rao et al. 2003). One of the most significant risk factors is preoperative cardiogenic shock. Extracorporeal membrane oxygenation (ECMO) is often used to improve and stabilize the preoperative condition of LVAD patients. However, ECMO support prior to LVAD implantation has recently been reported to significantly worsen survival rates (Toda et al. 2012). ECMO has also been associated with systemic inflammatory response (SIRS) (Chen et al. 2013; Mc et al. 2010). Hence, we hypothesized that patients who receive ECMO support prior to LVAD implantation need higher doses of inotropes than those without ECMO. The aim of this study was to elucidate the difference in inotrope use between patients who receive ECMO support prior to LVAD implantation and those who do not using the vasoactive inotropic score (VIS).

## Methods

Approval for this study was obtained from the ethics committee at our institution, which waived the requirement for written informed consent from each patient because the retrospective registry involves no additional risk to the subjects. We retrospectively investigated 108 patients with advanced heart failure who underwent LVAD implantation as a bridge to heart transplantation at the National Cerebral and Cardiovascular Center between May 1999 and September 2011. We excluded two patients who underwent right ventricular assist device implantation. Four patients whose ECMO could not be removed during weaning from cardiopulmonary bypass (CPB) were excluded as well because the cardiac index (CI) and mixed venous oxygen saturation (SvO2) would be inaccurate in these patients. Anesthesia was induced with midazolam and fentanyl, and rocuronium was given to facilitate orotracheal intubation with a cuffed tube. Anesthesia was maintained in all patients with propofol and fentanyl or remifentanil. All patients were catheterized with Swan-Ganz catheters capable of automatically and continuous measurement of cardiac output and SvO2. Inotropes, vasopressors, and vasodilators are administered at the discretion of the individual attending anesthesiologist, depending on the hemodynamics. The target MAP was around 60–70 mm Hg, and the target CI was 2.0–2.5. There were 27 patients who received ECMO support prior to LVAD implantation. ECMO consisted of a membrane oxygenator and a centrifugal pump (Capiox, Terumo, Tokyo, Japan). We defined these 27 patients as the ECMO group, and the other 81 patients as the non-ECMO group.

The patients’ clinical data were collected from clinical records including demographics, preoperative laboratory data, and intraoperative variables. CI, mean arterial pressure (MAP), systemic vascular resistance index (SVRI), SvO2, and VIS were recorded at weaning from CPB, 30 min after weaning from CPB (min after CPB), 60 min after CPB, and at the end of the surgery. MAP and VIS were also recorded before induction of anesthesia (baseline).

We used a modification of the VIS described by Gaies et al. (2010). We expanded this formula to include the inotrope olprinone, which is a phosphodiesterase 3 inhibitor widely used in Japan; we chose 25 as its coefficient because the ratio of maintenance infusion of milrinone (0.25 µg/kg/min) was compared with that of olprinone (0.1 µg/kg/min) in a previous report (Orime et al. 1999). Additionally, Milrila^®^K (Astellas, Tokyo, Japan) specifies a maintenance dose range of 0.25–0.75 µg/kg/min, whereas Coretec^®^ (Eisai, Japan) lists the range as 0.1–0.3 µg/kg/min; therefore, we concluded that a coefficient for olprinone 2.5 times that of milrinone was appropriate.

The modified VIS was defined as: (dopamine µg/kg/min × 1 + dobutamine µg/kg/min × 1 + epinephrine µg/kg/min × 100 + noradrenaline µg/kg/min × 100 + milrinone µg/kg/min × 10 + olprinone µg/kg/min × 25).

### Types of LVAD and surgical technique

All surgical procedures were performed through a median sternotomy during CPB. The outflow cannula was anastomosed to the ascending aorta and the inflow cannula to the left ventricular apex, without arresting the heart, to minimize ischemic insult to the right ventricle. The LVAD was then placed between the inflow and outflow cannulae (Toda et al. 2012). The LVADs comprised 91 paracorporeal devices (Toyobo-VAS: Nipro), and 17 implantable devices (two DuraHeart [Terumo]; two HeartMate VE [Thoratec]; four Novacor [World Heart]; seven Evaheart [Sun Medical]; one Jarvik 2000 [Jarvik Heart]; and one HeartMate II [Thoratec]).

### Statistical analysis

To determine the required sample size, we estimated the expected difference in the mean VISs as eight, and the expected standard deviation as 12. A power of 0.8 and an α of 0.05 were used to determine that 24 patients in the ECMO group would be appropriate. Hemodynamic data, VIS, and each inotrope dose were subjected to repeated-measures ANOVA. If the difference between the groups was significant, an independent *t* test was used to determine the difference at each time point.

Statistical significance was set at a level of 0.05. All statistical analyses were performed with EZR (Saitama Medical Center, Jichi Medical University, Saitama, Japan), which is a graphical user interface for R (R Foundation for Statistical Computing, Vienna, Austria). More precisely, it is a modified version of an R commander designed to add statistical functions frequently used in biostatistics (Kanda 2013).

## Results

Table 1 shows the demographic data of all patients. Preoperative variables and intraoperative characteristics in the ECMO group and the non-ECMO group are shown in Table 2. There were no significant differences between groups in sex, age, body surface area, body mass index, aspartate aminotransferase, alanine aminotransferase, serum B-type natriuretic peptide level, anesthesia time, operation time, CPB time, and blood loss. However, a larger number of patients in the ECMO group required preoperative intra-aortic balloon pump support, mechanical ventilation, and intraoperative nitric oxide use. Serum total bilirubin, serum creatinine, and serum blood urea nitrogen (BUN) were significantly higher in the ECMO group. White blood cell count and C-reactive protein levels before LVAD implantation were significantly higher in the ECMO group. Hemoglobin, platelet count, serum total protein, and albumin levels were significantly lower in the ECMO group.

**Table 1:** Preoperative patient characteristics

Characteristic	Number or mean ± SD
Sex (M/F)	76/32
Age (year)	34.6 ± 12.8
BSA (m^2^)	1.55 ± 0.20
BMI (kg/m^2^)	19.4 ± 3.7
Serum total bilirubin (mg/dl)	2.3 ± 2.3
Serum creatinine (mg/dl)	1.4 ± 1.1
Serum BNP (pg/ml)	1436 ± 994
Preoperative IABP support	64 (59.3)
Preoperative ECMO	27 (25.0)
LVEDD (mm)	73 ± 11
LVESD (mm)	66 ± 11
LVEF (%)	17 ± 9

Data are presented as mean ± SD, or number (%)

*BSA* body surface area, *BMI* body mass index, *BNP* brain natriuretic peptides, *IABP* intra-aortic balloon pump, *ECMO* extracorporeal membrane oxygenation, *LVEDD* left ventricular end-diastolic diameter, *LVESD* left ventricular end-systolic diameter, *LVEF* left ventricular ejection fraction

**Table 2:** Preoperative variables and intraoperative characteristics in both groups

	ECMO group (*n* = 27)	Non-ECMO group (*n* = 81)	*p* value
Sex (male)	19 (70.4)	57 (70.4)	1.000
Age (year)	33.4 ± 12.4	35.0 ± 13.0	0.573
BSA (m^2^)	1.53 ± 0.18	1.56 ± 0.21	0.593
BMI (kg/m^2^)	18.6 ± 2.7	19.6 ± 3.9	0.216
Preoperative IABP support	24 (88.9)	40 (49.4)	<0.001
Preoperative mechanical ventilation	22 (81.5)	7 (8.6)	<0.001
White blood cell count(/ml)	8580 ± 2961	10,789 ± 4706	0.006
Hemoglobin (g/dl)	10.2 ± 1.8	11.3 ± 1.9	0.009
Platelet count (x10^4^/ml)	13.0 ± 7.9	24.4 ± 24.4	0.022
C-reactive protein (mg/dl)	6.1 ± 4.6	3.1 ± 3.3	<0.001
Serum total bilirubin (mg/dl)	3.7 ± 3.8	1.9 ± 1.3	<0.001
Serum total protein (g/dl)	6.0 ± 0.7	6.4 ± 0.7	0.015
Serum albumin (g/dl)	3.3 ± 0.7	3.6 ± 0.5	0.010
AST (IU/L)	243 ± 507	124 ± 315	0.157
ALT (IU/L)	182 ± 350	178 ± 392	0.959
Serum creatinine (mg/dl)	1.9 ± 1.8	1.2 ± 0.7	0.006
Serum BUN (mg/dl)	40 ± 26	29 ± 19	0.024
Serum BNP (pg/ml)	1380 ± 985	1453 ± 1003	0.772
Anesthesia time (min)	522 ± 140	511 ± 169	0.767
Operation time (min)	423 ± 132	398 ± 151	0.444
CPB time (min)	161 ± 60	156 ± 59	0.689
NO use in operation	16 (59.3)	25 (30.9)	0.008
Blood loss (ml)	3054 ± 3010	2177 ± 2188	0.114

Data are presented as mean ± SD, or number (%)

*ECMO* extracorporeal membrane oxygenation, *BSA* body surface area, *BMI* body mass index, *IABP* intra-aortic balloon pump, *AST* aspartate aminotransferase, *ALT* alanine aminotransferase, *BUN* blood urea nitrogen, *BNP* brain natriuretic peptides, *CPB* cardiopulmonary bypass, *NO* nitric oxide

**Fig. 1:**
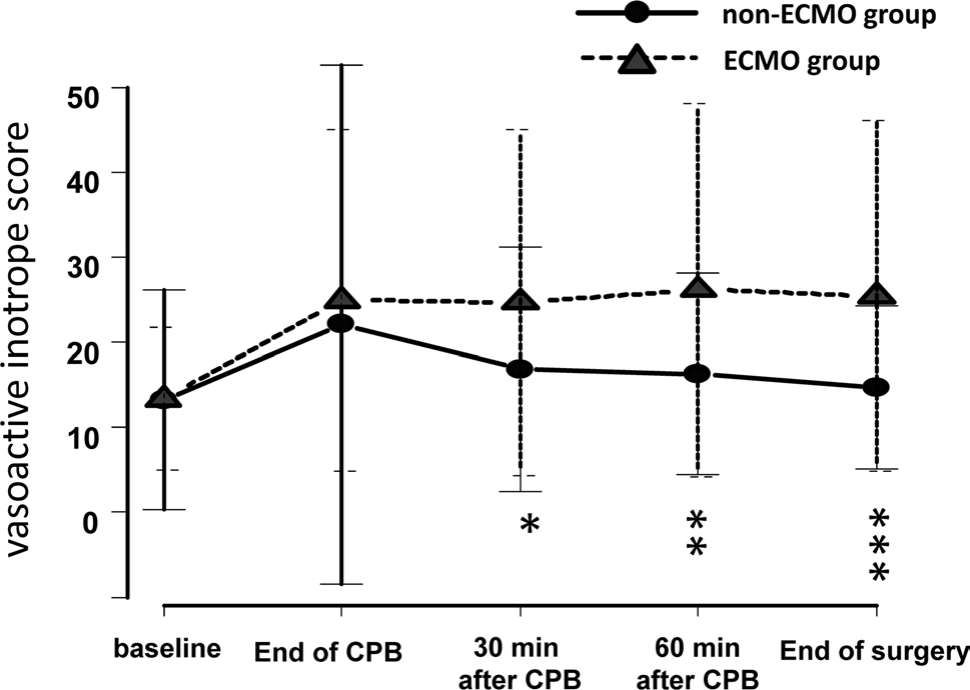
Change in vasoactive inotrope score during left ventricular assist device implantation in patients who received extracorporeal membrane oxygenation (ECMO) compared with those who did not. All data are expressed as mean (*filled symbols*) ± SD (*bars*). The *filled circles* and *solid line* represents the non-ECMO group, and the *filled triangles* and *dotted line* represents the ECMO group. ECMO extracorporeal membrane oxygenation, CPB cardiopulmonary bypass. *p < 0.05, **p < 0.01, ***p < 0.001

**Fig. 2:**
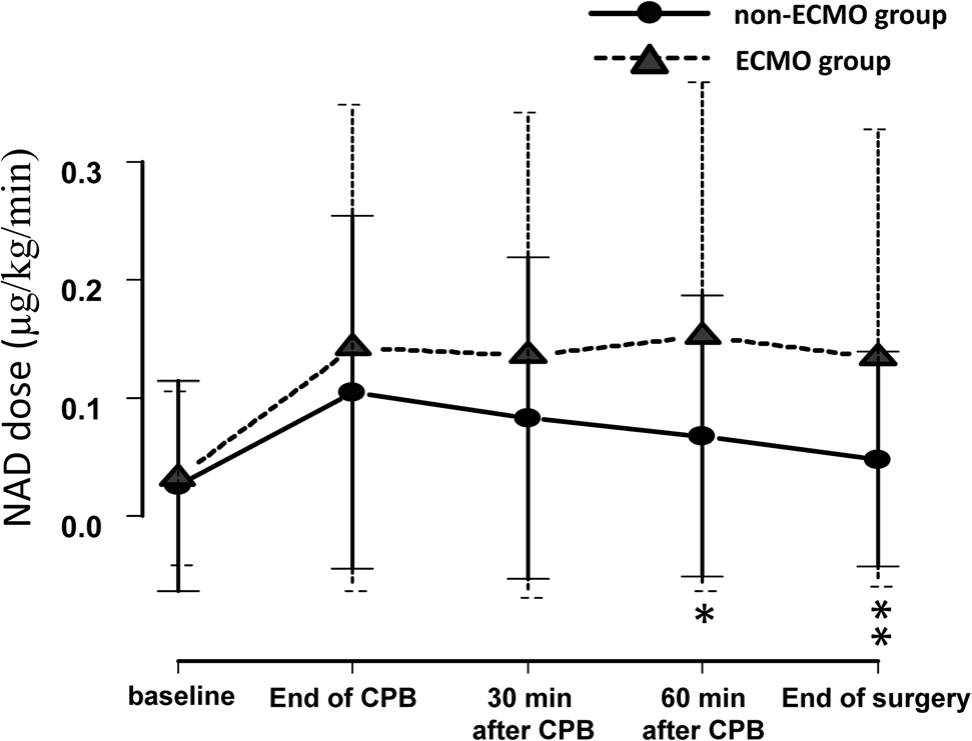
Change in noradrenaline dose required during left ventricular assist device implantation in patients who received extracorporeal membrane oxygenation (ECMO) compared with those who did not. All data are expressed as mean (*filled* *symbols*) ± SD (*bars*). The *filled circles* and *solid line* represents the non-ECMO group, and the *filled triangles* and *dotted line* represents the ECMO group. ECMO extracorporeal membrane oxygenation, NAD noradrenaline, CPB cardiopulmonary bypass. *p < 0.05, **p < 0.01

There was no significant difference between the group in terms of baseline MAP (70.6 ± 15.0 mm Hg in the ECMO group, 67.8 ± 14.0 mm Hg in the non-ECMO group, *p* = 0.395). The hemodynamic changes in both groups are shown in Table 3. There were no significant differences between the ECMO group and the non-ECMO group in terms of hemodynamic parameters such as MAP, CI, SVRI, and SvO_2_. However, changes in the VIS were significantly different between groups (Table 4, *p* = 0.030). Each dose of inotrope was compared by repeated-measures ANOVA, which revealed that only the noradrenaline dose was significantly different between the groups (*p* = 0.044). Figure 1 shows the VIS for both groups; an independent *t*-test revealed that the VIS was significantly higher in ECMO group at 30 min after CPB (*p* = 0.03), 60 min after CPB (*p* = 0.003), and at the end of the surgery (*p* < 0.001). Figure 2 shows the noradrenaline dose for both groups during LVAD implantation surgery; an independent *t*-test revealed that the doses of noradrenaline were significantly higher in ECMO group at 60 min after CPB (*p* = 0.013), and at the end of surgery (*p* = 0.002).

**Table 3:** Hemodynamic changes in both groups

	Group	End of CPB	30 min after CPB	60 min after CPB	End of surgery	*p* value^a^
MAP (mm Hg)	Non-ECMO group	66.4 ± 11.5	70.7 ± 12.2	72.5 ± 9.8	74.1 ± 16.2	0.295
	ECMO group	68.8 ± 10.1	68.3 ± 13.2	68.4 ± 13.4	70.1 ± 11.5	
CI (L/min/m^2^)	Non-ECMO group	2.5 ± 0.9	2.4 ± 1.1	2.5 ± 0.8	2.6 ± 0.6	0.989
	ECMO group	2.2 ± 1.3	2.4 ± 0.6	2.4 ± 0.5	2.6 ± 0.6	
SVRI (dynes s/cm^5^/m^2^)	Non-ECMO group	2120 ± 945	2139 ± 822	2214 ± 803	2082 ± 777	0.808
	ECMO group	2489 ± 844	2197 ± 670	2184 ± 898	1962 ± 518	
SvO_2_ (%)	Non-ECMO group	78.2 ± 8.7	75.3 ± 13.3	74.8 ± 7.5	73.8 ± 8.3	0.120
	ECMO group	73.6 ± 13.7	70.0 ± 12.2	69.9 ± 9.2	68.2 ± 8.4	

Data are presented as mean ± SD

*CPB* cardiopulmonary bypass, *MAP* mean arterial pressure, *CI* cardiac index, *SVRI* systemic vascular resistance index, *SvO*_*2*_ mixed venous oxygen saturation, *ECMO* extracorporeal membrane oxygenation

^a^compared between the two groups

**Table 4:** Change in vasoactive inotrope score and catecholamine dose in both groups

	Group	Baseline	End of CPB	30 min after CPB	60 min after CPB	End of surgery	*p* value^a^
VIS	Non-ECMO group	13.2 (5.7–16.0)	22.1 (9.4–23.0)	16.8 (8.7–20.0)	16.3 (9.5–17.5)	14.7 (8.8–16.6)	0.030
	ECMO group	13.4 (8.1–16.0)	24.9 (12.7–26.4)	24.7 (12.1–25.3)	26.1 (12.3–33.3)	25.5 (12.7–31.7)	
DOA (µg/kg/min)	Non-ECMO group	3.3 (0–5.0)	3.8 (3.0–5.0)	3.8 (3.0–5.0)	3.8 (3.0–5.0)	3.8 (3.0–5.0)	0.250
	ECMO group	3.4 (1.8–4.9)	4.1 (3.0–5.0)	4.3 (3.3–5.0)	4.2 (3.0–5.0)	4.0 (3.0–5.0)	
DOB (µg/kg/min)	Non-ECMO group	5.3 (3.5–7.0)	2.0 (0–3.5)	2.2 (0–3.9)	2.2 (0–4.0)	2.6 (0–4.4)	0.908
	ECMO group	5.0 (3.8–6.5)	2.2 (0–4.5)	2.3 (0–4.8)	2.2 (0–4.3)	2.5 (0–4.3)	
NAD (µg/kg/min)	Non-ECMO group	0.02 (0–0)	0.10 (0–0.12)	0.08 (0–0.1)	0.07 (0–0.08)	0.05 (0–0.05)	0.044
	ECMO group	0.03 (0–0)	0.14 (0–0.13)	0.14 (0–0.15)	0.15 (0–0.17)	0.13 (0–0.18)	
AD (µg/kg/min)	Non-ECMO group	0.001 (0–0)	0 (0–0)	0.001 (0–0)	0.001 (0–0)	0.004 (0–0)	0.162
	ECMO group	0.006 (0–0)	0.004 (0–0)	0 (0–0)	0 (0–0)	0.013 (0–0)	
Milrinone (µg/kg/min)	Non-ECMO group	0.2 (0–0.4)	0.2 (0–0.4)	0.2 (0–0.4)	0.2 (0–0.4)	0.2 (0–0.4)	0.580
	ECMO group	0.1 (0–0.2)	0.2 (0–0.5)	0.2 (0–0.5)	0.2 (0–0.5)	0.2 (0–0.5)	
Olprinone (µg/kg/min)	Non-ECMO group	0.004 (0–0)	0.1 (0–0)	0.05 (0–0)	0.05 (0–0)	0.05 (0–0)	0.847
	ECMO group	0.003 (0–0)	0.1 (0–0)	0.10 (0–0.3)	0.10 (0–0.3)	0.08 (0–0.2)	

Data are presented as mean (1st quartile–3rd quartile)

*VIS* vasoactive inotrope score, *CPB* cardiopulmonary bypass, *DOA* dopamine, *DOB* dobutamine, *NAD* noradrenaline, *AD* adrenaline

^a^compared between the non-ECMO group and the ECMO group

## Discussion

Patients who received ECMO support prior to LVAD implantation required significantly more vasopressor support compared with those who did not receive preoperative ECMO support. This was most likely caused by the patients’ low systemic vascular resistance concomitant with the occurrence of SIRS during ECMO (Mc et al. 2010).

Although the pathophysiology is not completely understood, previous studies have revealed that cardiac surgery using CPB induces SIRS (Cremer et al. 1996; Laffey et al. 2002; Delannoy et al. 2009). Inflammatory mediators are activated and released from blood cells as a result of exposure to the artificial surfaces of the extracorporeal circuit, surgical trauma, hypothermia, and tissue ischemia–reperfusion (Belhaj 2012; Kozik and Tweddell 2006). Cytokines play an important part in the inflammatory reaction caused by surgical trauma; they mediate the local inflammatory response, resulting in systemic changes (Gozdzik et al. 2014).

Although there are some pathophysiological differences between CPB and ECMO, almost all patients treated with ECMO are associated with SIRS, which is characterized by a “cytokine storm”, leukocyte activation, and multisystem organ dysfunction (Mc et al. 2010; Chen et al. 2013). An animal model study revealed that animals given ECMO support develop tachycardia and hypotension within 1–2 h of ECMO initiation (Mc et al. 2010).

In our study, patients who received preoperative ECMO support needed more vasopressor to maintain normal MAP levels. This may indicate that hypotension is enhanced by the synergetic effect of the cytokine storm caused by ECMO and CPB. The baseline VIS (before induction of anesthesia) was not different between the groups; therefore, the synergetic effect of both ECMO and CPB may have decreased systemic vascular resistance, which resulted in more vasopressor being required to maintain blood pressure. In the current study, serum total bilirubin, serum creatinine, and serum BUN were significantly higher in the ECMO group; this may reflect that patients who required ECMO support did not recover from preoperative end-organ dysfunction because of cardiogenic shock. Preoperative white blood cell count and serum C-reactive protein level were also significantly higher in the ECMO group, suggesting that preoperative SIRS induced by ECMO may have an impact on hemodynamics. Bertrand et al. investigated the association between biological markers and CPB-induced SIRS. They found that baseline C-reactive protein is significantly higher in patients with SIRS than in patients without SIRS (6.7 vs 1.8 mg/l, *p* = 0.016) (Delannoy et al. 2009). This agrees with our result in which the baseline C-reactive protein was significantly higher in the ECMO group, who needed more vasopressor because of SIRS induced by ECMO and CPB.

In our study, inotropes, vasopressors, and vasodilators were administered at the discretion of the individual attending anesthesiologist, depending on the hemodynamics and real-time transesophageal echocardiography. However, there was no difference between the groups in terms of the hemodynamic parameters such as MAP, CVP, SVRI, and SvO2. It follows that the target hemodynamics were the same in both groups. Each attending anesthesiologist used noradrenaline as a vasopressor to maintain systemic vascular resistance within normal range. However, we were unable to determine whether this method of vasopressor use improved the outcome of the patients who had received preoperative ECMO support. Few studies to date have demonstrated a significant survival benefit of one vasopressor over another. Further study is necessary to demonstrate that the use of a vasopressor can improve the outcome of patients after LVAD implantation.

There were some limitations to this study. First, the number of patients was relatively small. We conducted a power analysis to determine the minimum number required for each group. Because no previous studies comparable to ours have been conducted, we empirically designated the difference in the VIS means as eight. The variance of VIS was computed based on the VIS variance of the first 20 patients. Although we conducted a power analysis, it is possible that the ideal sample size is larger than presented here because of the empirical process. Second, this study was retrospective and the accuracy of chart documentation cannot be guaranteed. Besides, we cannot eliminate the other multiple factors affecting vasodilatory responses other than ECMO, including multi-organ failure, use of IABP, mechanical ventilation, possibly intraoperative blood loss or dosage of phosphodiesterase inhibitor. Third, the coefficient of olprinone in modified VIS may be inaccurate because it was defined based on the study with small number of patients. Finally, this was a single-center study, and thus our findings may not be generalizable to other patient populations.

## Conclusions

In conclusion, patients supported by ECMO prior to LVAD implantation required significantly more noradrenalineto maintain normal hemodynamic parameters compared with patients without ECMO.
